# Isolated spontaneous biceps abscess causing septic shock in a diabetic patient: A rare case report

**DOI:** 10.1016/j.ijscr.2020.07.075

**Published:** 2020-08-07

**Authors:** M Masood Sidiqi, Benjamin Witte

**Affiliations:** Kalgoorlie Regional Hospital, Western Australia, Australia

**Keywords:** Biceps, Brachii, Abscess, Sepsis

## Abstract

**Introduction:**

Intramuscular abscesses, particularly in the biceps brachii, are an extremely rare phenomenon. When present they are usually secondary to trauma, intramuscular injections, or systemic disease.

**Presentation of case:**

A 56 year old diabetic woman presented to our emergency department with a 3 day history of fever, cough, and a painful left shoulder. Although she had a mechanical fall 2 weeks prior, she denied any pain in her shoulder immediately after the fall. She also denied any history of drug abuse or recent intramuscular injection. On examination she looked acutely unwell and was in acute septic shock requiring inotropic support. Computed tomography of her shoulder showed a large intramuscular abscess in her left biceps brachii muscle. She was immediately taken to the operating theatre for open exploration and washout of the abscess. The multiloculated abscess was tracking into the glenohumeral joint. Post operatively she showed significant clinical improvement and after a 2 week course of intravenous antibiotics recovered well and was discharged from hospital.

**Discussion:**

Intramuscular abscesses are usually seen in patients who are immunocompromised. Intramuscular needle injections and haematomas secondary to trauma are also risk factors. To the best of our knowledge, there have only been 4 published reports in the English literature of intramuscular abscess formation in the biceps brachii.

**Conclusion:**

We report a case of a seemingly spontaneous intramuscular biceps abscess in a diabetic patient presenting with septic shock.

## Highlights

• Intramuscular abscesses, particularly in the biceps brachii, are very rare.
• The majority are associated with haematoma secondary to trauma, intramuscular injections, or systemic disease.
• If untreated, it can rapidly lead to septic shock and involvement of surrounding joints.

## 1 Introduction

Abscess formation within the biceps brachii muscle is an exceedingly rare condition. Few case reports of spontaneous muscle abscesses, particularly in the biceps, are present in the literature. To the best of our knowledge, there have only been 4 published reports in the English literature of intramuscular abscess formation in the biceps brachii. All of these were associated with either intramuscular injections, traumatic haematoma formation, or systemic conditions such as Tuberculosis and Gonococcal disease [[1], [2], [3], [4]]. We report a case of a diabetic patient who presented with septic shock secondary to a seemingly spontaneous biceps abscess. The work has been reported in line with the SCARE criteria [5].

## 2 Presentation of case

A 56-year old indigenous Australian woman presented at our hospital acutely unwell with fever, cough, and left shoulder pain. She was brought in from the tribal lands by her family complaining she was unable to sleep at night, had an occasional cough, and purulent nasal discharge. She was also complaining of left shoulder pain for the last 3 days. This was on a background of having a mechanical fall two weeks prior, although the patient confirmed that she was able to move the shoulder without any pain after the fall. Her past medical history revealed poorly controlled type 2 diabetes, hypertension, hypothyroidism, and a seizure disorder secondary to a mild traumatic head injury. She was a non-smoker and had no history of drug abuse. Her medications included aspirin, metformin, ramipril, sertraline, thyroxine, and levetiracetam. There was also no history of any intramuscular injection in the recent past. On examination she was febrile at 38.2, tachycardic and hypotensive with a systolic blood pressure of 90. Her left upper arm and shoulder were tender and mildly swollen. She had reduced range of motion in the shoulder joint but there was no obvious fluctuant collection clinically evident. There was no laceration or bite mark visible and her left upper limb was neurovascularly in-tact.

**Fig. 1:**
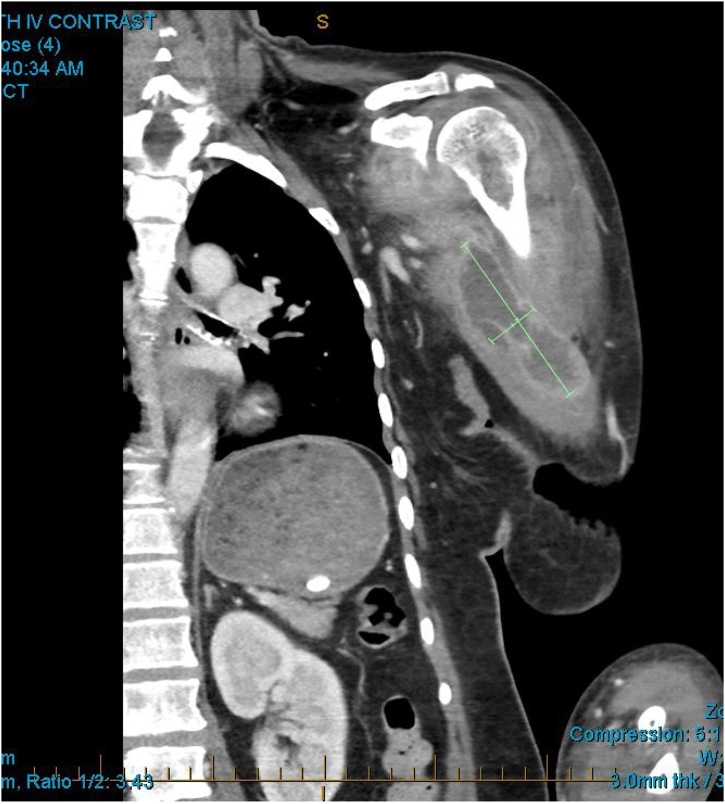
Computed tomography showed a large intramuscular abscess in the biceps brachii.

**Fig. 2:**
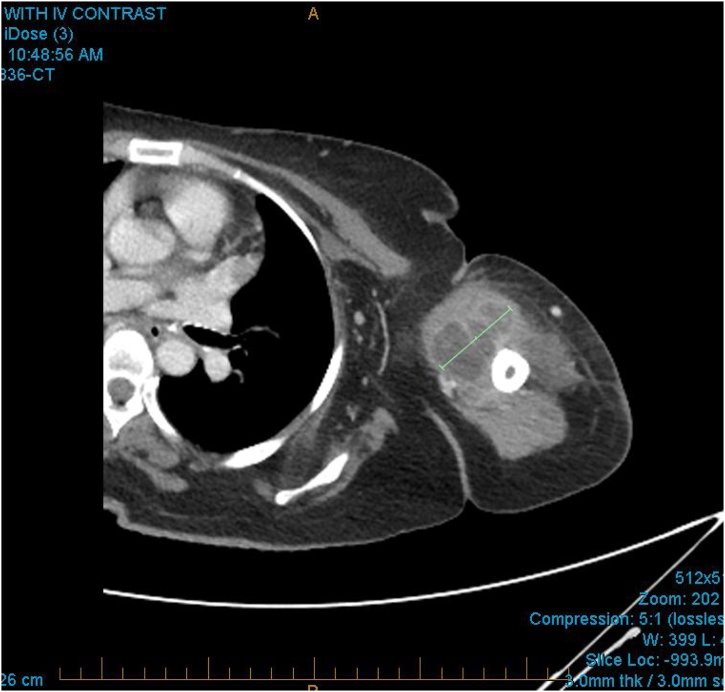
The abscess was multi-loculated.

X-ray of the left shoulder and arm were unremarkable. The patient’s inflammatory markers were elevated with a white cell count of 16.1 × 10^9^/L (normal 4–11 × 10^9^/L) and a C reactive protein of 210 mg/L (normal <5 mg/L). Her other blood tests including haemoglobin, creatinine, liver function, lipase and troponin were within normal limits. Although she was initially admitted under the general physicians for sepsis of unknown origin and put on broad spectrum antibiotics, she continued to deteriorate requiring vasopressor support in the high dependency unit. She subsequently had a CT scan of her left shoulder which showed a multi-loculated abscess in the left biceps muscle measuring approximately 45 × 80 × 27 mm (Fig. 1, Fig. 2).

The patient was immediately taken to the operating theatre where she had an open exploration and washout of the left shoulder. After reviewing the CT imaging to plan our approach, we made an incision over the delto-pectoral groove and extended down to the upper arm where we gained access to the abscess. Approximately 20 mls of purulent fluid was drained from the biceps. There were two moderately sized locules of pus within the muscle and there was a small amount of purulent fluid tracking into the gleno-humeral joint. We bluntly dissected with our finger to open up all the loculations and the infected tissue planes. We then performed an open mini-arthrotomy of the glenohumeral joint and drained <5mls of purulent fluid. The rest of the muscle and fascia looked healthy. After copious washout, two Yates drains were left within the biceps brachii and the wound was partially closed. Post operatively she was sent to a tertiary centre under the care of the Orthopaedic surgeons, where she improved significantly both clinically and biochemically. The patient was weaned off inotropic support and her C reactive protein fell to less than 50. Intraoperative specimens grew Streptococcus bovis (sub gallolyticus) and she completed 2 weeks of intravenous Ceftriaxone as per the advice of the local Microbiologist. Her shoulder pain improved significantly, she regained most of her range of motion, and was subsequently discharged from hospital.

## 3 Discussion

The biceps brachii muscle is an infrequent location of abscess formation, and the diagnosis is often not straightforward. The classical signs of abscess may be lacking because of the overlying muscle and tense fascia. In such circumstances needle aspiration may confirm the diagnosis, but in our case, given the patient’s haemodynamic instability, immediate surgical drainage was performed. Predisposing conditions include immunocompromised states such as diabetes, malignancy, intravenous drug use, or HIV [6]. There have also been isolated reports of patients with gonococcal septicaemia and tuberculosis presenting with an abscess in their biceps [1,4]. Haematoma due to arm trauma or intramuscular needle injections are also potential sources of infection [2,3]. However, as in the present case, patients may present without any underlying direct aetiology.

Interestingly, the patient grew Streptococcus bovis (sub gallolyticus) in the intra-operative pus specimens that were cultured. This is a Gram positive bacteria which is classified as a non-enterococcal Streptococcus in Lancefield's group D and is the pathogen agent of several types of infection including urinary tract infections, septicemia and endocarditis, but also unusual presentations such as endophthalmitis and soft tissue abscess, amongst others [8,9]. Although several unusual bacterial infections have been recognised in relationship with neoplastic lesions of the colon, Streptococcus bovis has the strongest and best documented association with colonic cancer. The published literature suggests that all types of Streptococcus bovis infection mandates complete gastrointestinal screening and, if negative, endoscopic follow-up [8,10]. Unfortunately for our patient, who is an indigenous person from the remote tribal lands, it was very difficult to subsequently contact her and organise outpatient follow up despite our best efforts (although this is ongoing).

To date, only four cases of biceps intramuscular abscess have been reported. Frye et al. [2] described a case of a young patient who ruptured his distal biceps tendon, only to present seventy-two hours later with a spontaneously draining abscess over his anterior distal humerus. Dunn [3] presented a case of pyogenic biceps abscess in a professional bodybuilder who was injecting anabolic steroids and a mixture of fatty acids intramuscularly. Furthermore Chapple et al. [7] reported a case of infected hematoma after pectoralis major rupture. However the patient only showed signs and symptoms of infection 7 weeks after the injury when he presented with increased swelling, fever, and weight loss. Our patient presented two weeks post sustaining a fall from her wheelie walker. One possible explanation is that she sustained an occult asymptomatic haematoma of her arm which subsequently became infected. However this is unlikely, given the absence of pain in her arm immediately after the fall and the fact that purulent fluid was exclusively drained during surgical evacuation without any evidence of haematoma. Furthermore she was not on any anticoagulant medications that would predispose her to spontaneous haematoma formation. This patient also did not grow anything on her blood cultures and was not bacteraemic, suggesting this was haematogenous in origin. The exact cause of the abscess in our case remains unclear.

## 4 Conclusion

Soft tissue infections should always be considered in patients with sepsis of unknown origin, especially if they are immunocompromised. Although abscess of the biceps muscle is very rare, this case highlights the importance of careful clinical examination of the extremities in such patients.

## Conflict of interest

Authors have no conflict of interest to disclose.

## Sources of funding

None.

## Ethical approval

Ethical approval is not applicable.

## Consent

Written consent was obtained from the patient for publication of this case report and accompanying images. A copy of the written consent is available for review by the Editor-in-Chief of this journal on request.

## Author contribution

Dr Masood Sidiqi contributed in medical record review, literature search, and writing of the draft. Dr Benjamin Witte contributed towards review of the paper.

## Guarantor

All authors have read and approved the manuscript and accept full responsibility for the work.

## Provenance and peer review

Not commissioned, externally peer-reviewed.
